# Malignant perivascular epithelioid cell tumor (PEComa) of the uterus

**DOI:** 10.1186/s12905-022-02119-9

**Published:** 2022-12-16

**Authors:** Bo Cao, Yi Huang

**Affiliations:** Department of Gynecology Oncology, Hubei Cancer Hospital, Tongji Medical College, Huazhong University of Science and Technology, Hubei, China

**Keywords:** Perivascular epithelioid cell tumor (PEComa), Uterus neoplasm, Tuberous sclerosis complex (TSC), Human melanoma black 45 (HMB-45)

## Abstract

**Background:**

Perivascular epithelioid cell tumors (PEComas) of the uterus is a rare type of mesenchymal tumors associated with myelomelanocytic differentiation and distinctive histological appearances. So far, the reported cases of uterine PEComas are usually benign. Documented malignant cases with aggressive behavior appear to be less common.

**Case presentation:**

We report a 37-year-old female who received abdominal hysterectomy for uterine tumor in a local hospital. She was diagnosed with uterine leiomyosarcoma and referred to Hubei Cancer Hospital. Her histological slides were reviewed and immunohistochemical staining for specific markers of epithelial, melanocytic, myoid and some others were analyzed. The pathologic diagnosis was malignant uterine PEComa. Systematic imaging of the patient further revealed an abdominal para-aortic mass. She received pelvic and para-aortic lymph node dissection. Postoperative histology revealed para-aortic lymph nodal metastasis of malignant uterine PEComa. She received 8 cycles of chemotherapy after surgery. The chemotherapy regiment was epirubicin plus ifosfamide The patient is free of recurrence and metastasis 6 years after surgical resection.

**Conclusion:**

Uterine PEComas are indistinguishable from other uterine tumors such as leiomyoma and leiomyosarcoma before pathologic diagnosis could be made. For patients with malignant uterine PEComas, removal of both primary lesions and metastatic foci, if any, needs to be attempted. Postoperative chemotherapy or radiotherapy should also be considered in patients with distant metastases or positive lymph nodes.

## Background

The concept of perivascular epithelioid cell tumors (PEComas) was first proposed by Zamboni in 1996 to describe HMB-45-positive pancreatic tumors consisting of transparent cytoplasmic epithelial cells [1]. According to the WHO tumor classification, PEComas are a relatively rare group of mesenchymal tumors composed of epithelioid cells or sometimes mixed with spindle cells in variable proportions around small blood vessels. These tumors are characterized by a myelomelanocytic phenotype and often immunoreactive for melanocytic (HMB-45 and/or Melan-A) and smooth muscle (actin and/or desmin) markers [2]. PEComa is a heterogeneous and usually benign tumor that generally has a good prognosis. Malignant cases are rare and often present with local recurrence and distant metastasis [3].

PEComas may arise from various anatomic sites including the gynecological tract, in which the uterus is the most commonly affected organ. Fewer cases of gynecological PEComas are also reported in the cervix and even much rarer in the vagina, vulva, broad ligament, round ligament and ovary [4]. During the last decade, emerging case descriptions of uterine PEComas have highlighted a wide spectrum of their biological behaviors and morphological features. Nevertheless, more studies are still required to establish histological criteria to accurately classify the malignancy of uterine PEComas and to optimize their treatment strategies. It can be concluded that uterus PEComas are very rare tumors but need to be considered in the differential diagnosis of uterus lesions exhibiting unusual cytologic and immunohistochemical characteristics [5].

Currently, there is no standard treatment for neither primary nor recurrent and metastatic PEComa. Surgical resection is the first choice, and radical hysterectomy with bilateral salpingectomy is the best surgical method for the uterine PEComa [5].

This article reports a case of malignant uterine PEComa with para-aortic lymph nodal metastasis in a 37-year-old woman.

## Case presentation

A 37-year-old female patient was found to have an 8 × 7 × 7 cm uterine tumor by ultrasonography during examination for heavy menstrual bleeding and prolonged bleeding in a local hospital. She was initially diagnosed with uterine leiomyoma and underwent an abdominal hysterectomy and bilateral salpingectomy on October 10, 2015. The pathological findings from the local hospital suggested a diagnosis of uterine leiomyosarcoma. The patient was then referred to Hubei Cancer Hospital on November 3, 2015.

**Fig. 1:**
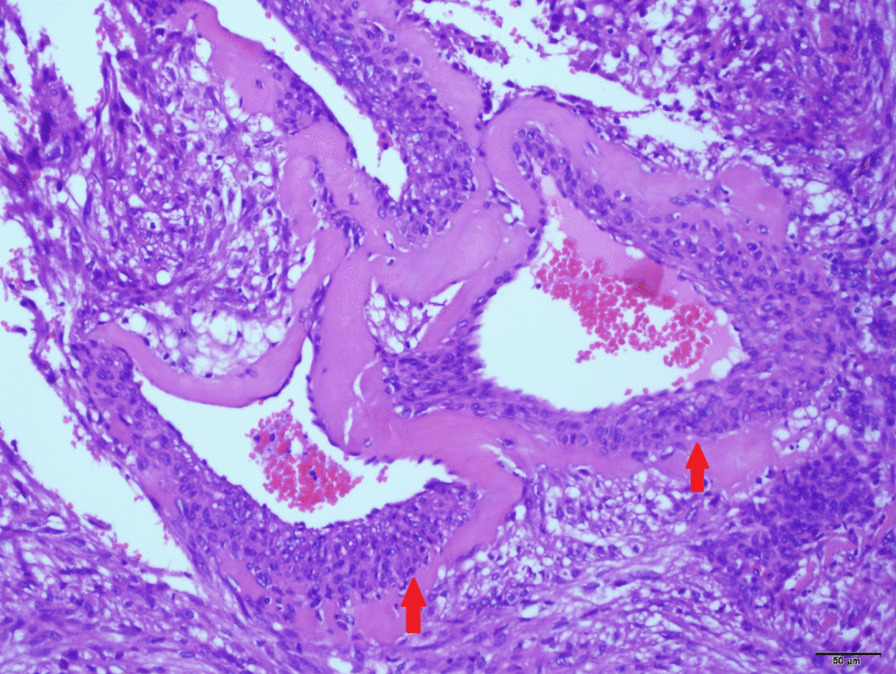
Microscopic appearance of the primary tumor cells (×200)

The pathological slides from the first surgery of the patient were reviewed with the Olympus BX-53 microscope. The pathological images were captured by the Olympus DP72 digital acquisition camera and no downstream processing was employed to enhance the resolution of the image. Microscopic examination revealed that the tumor was composed primarily of epithelioid cells and focal areas of spindle cells growing in a nested and alveolar pattern around small blood vessels (Figs. 1 and 200×).

**Fig. 2:**
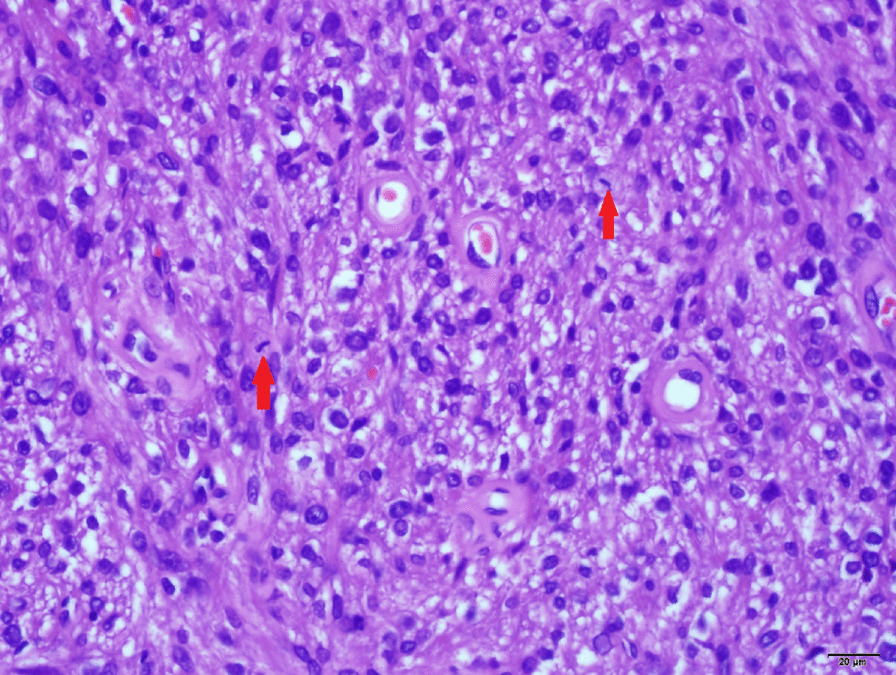
Pathological mitotic phase of the tumor cells (×400)

**Fig. 3:**
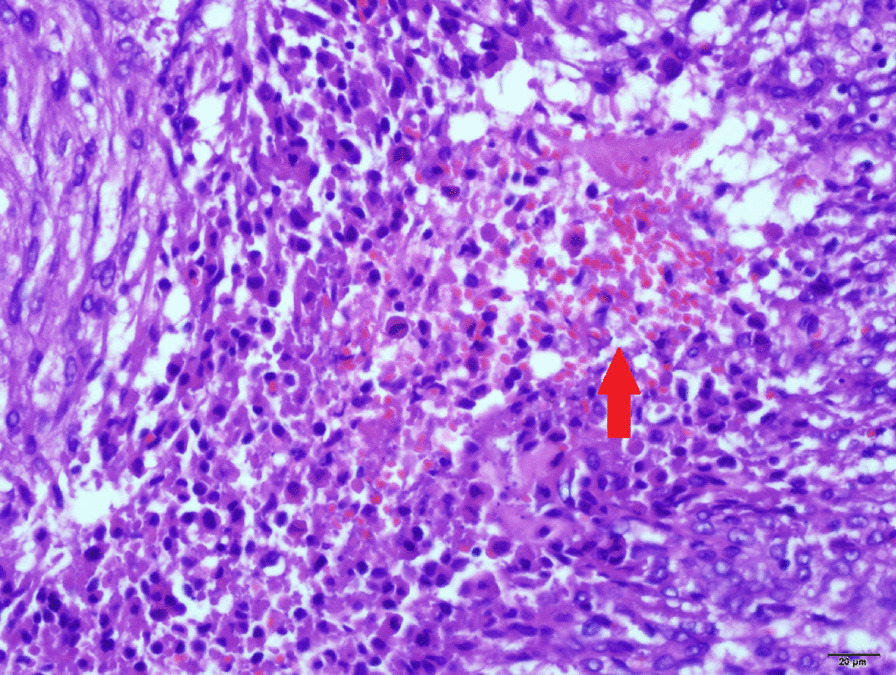
Large amount of tumor cell necrosis (×400)

**Fig. 4:**
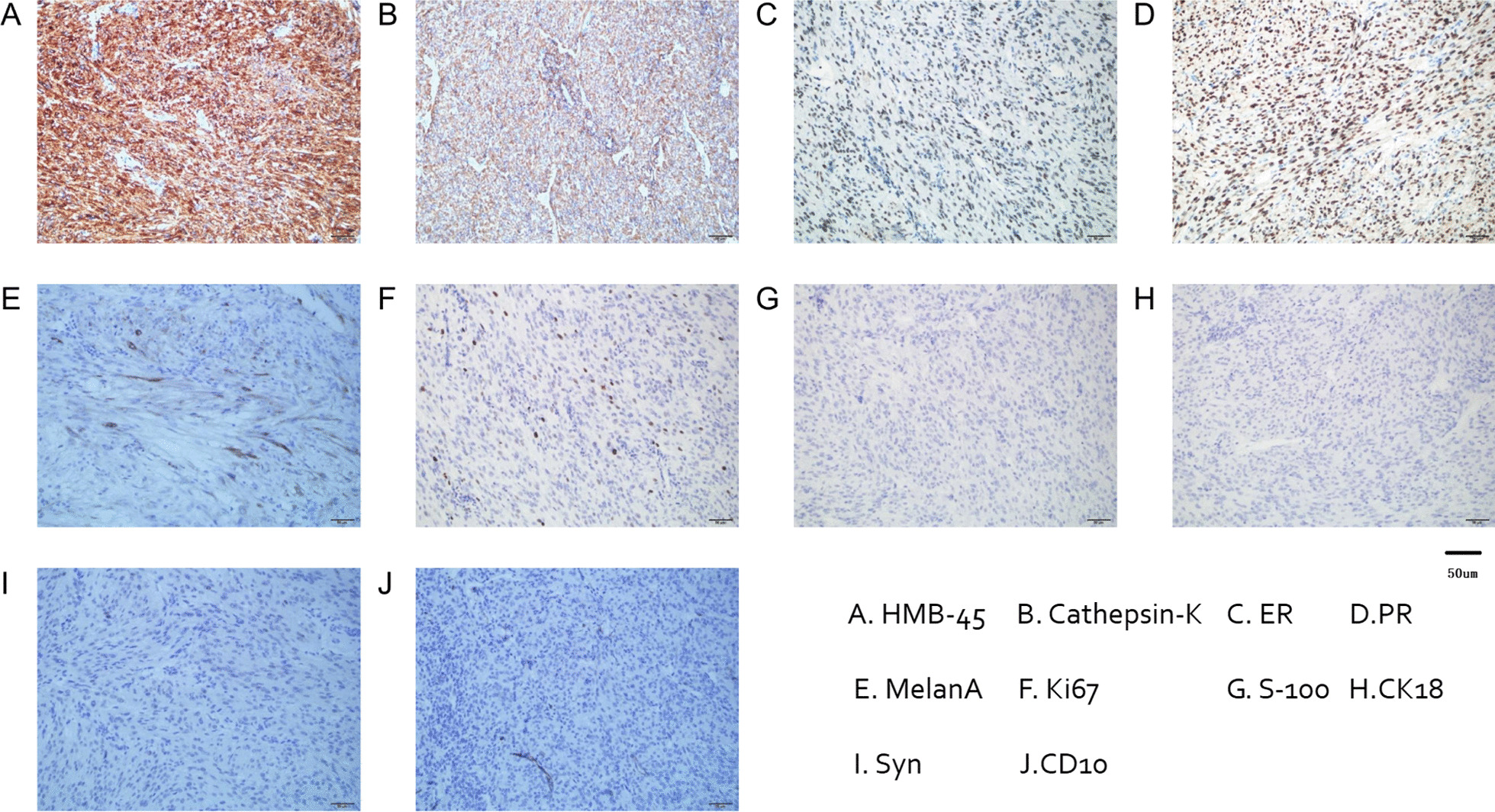
Immunohistochemical staining (×200)

The tumor cells had abundant cytoplasm varying from clear to focally granular and eosinophilic, centrally located round or oval nuclei, and inconspicuous nucleoli. Infiltrative growth patterns and mitotic figures were easily identified, whereas calcification or lipomatous components were not evident (Figs. 2 and 400×). In some areas a large amount of tumor cell necrosis was observed (Figs. 3 and 400×). Immunohistochemical staining of multiple markers further showed that the tumor cells were strongly and diffusely positive for HMB-45 and Cathepsin-K (both cytoplasmic) (Figs. 4 and 200×). While many tumor cells had strong nuclear staining of estrogen receptor (ER) and progesterone receptors (PR), some scattered tumor cells were positive for Melan-A in the cytoplasm. A proliferative index of 5% was also noted with Ki-67 nuclear labeling. By contrast, the tumor cells were negative for schwannoma marker (S-100), epithelial marker cytokeratin 18 (CK18), endocrine marker synaptophysin (Syn) and stromal cell marker CD10 (Figs. 4 and 200×). Taken together, these unique histological features and the immunohistochemical profile led us to a definite diagnosis of malignant uterine PEComa.

**Fig. 5:**
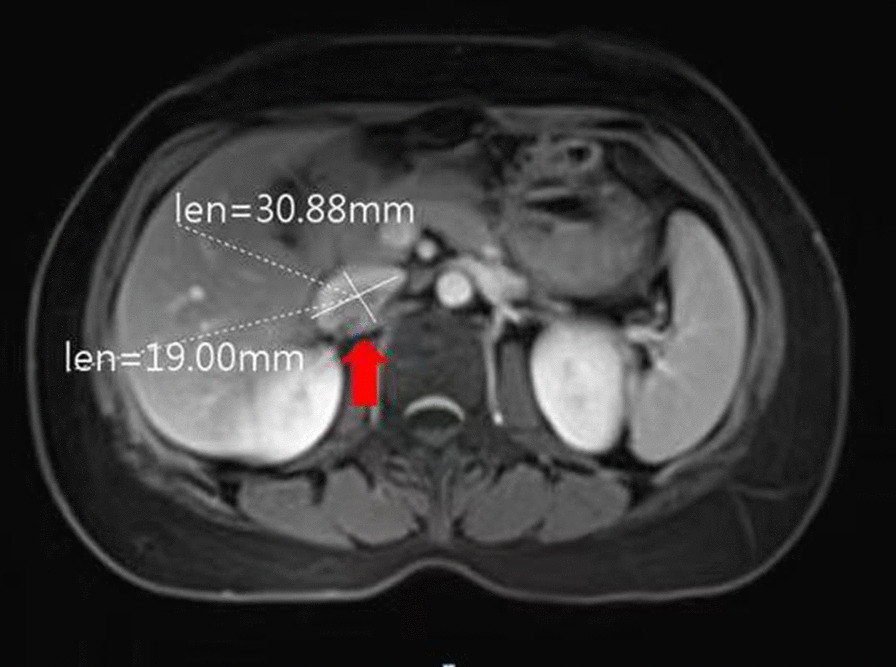
CT image of lymph node metastasis in the abdominal aorta

To further determine if there were local and/or distant metastases, we conducted a systematic imaging examination on the patient. The abdominal enhanced computed tomography (CT) scan revealed a solid, mixed-density mass of approximately 2.0 cm in diameter on the right side of the abdominal aorta which close to the right renal artery, indicating potential metastasis to the para-aortic lymph nodes (Fig. 5). No obvious abnormalities were found in the brain magnetic resonance imaging (MRI), chest CT and pelvic MRI.

**Fig. 6:**
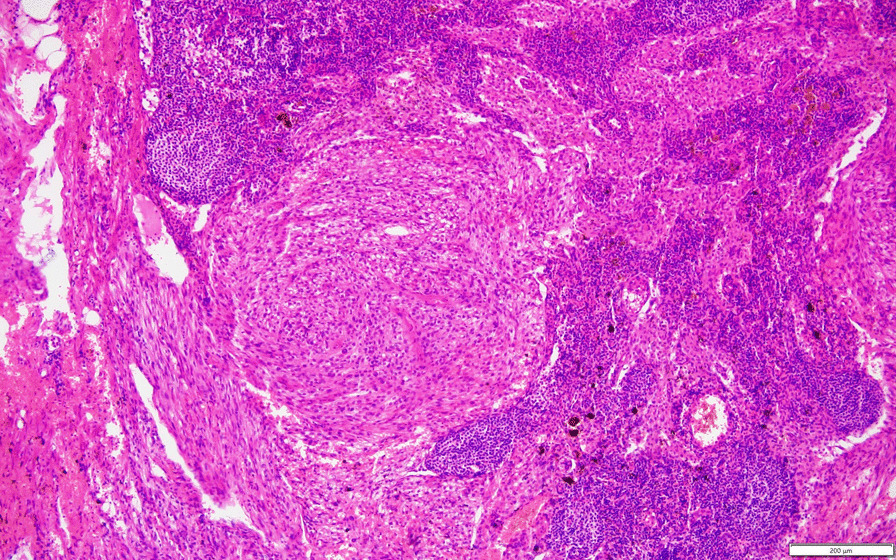
Microscopic appearance of the lymph node metastasis (40×)

Therefore on November 12, 2015, we performed the second surgery to dissect the bilateral pelvic lymph nodes and the abdominal aortic lymph nodes and bilateral ovary. Postoperative pathology showed metastasis to the abdominal aortic lymph nodes (3/11), whereas no metastatic lesions were found in the pelvic lymph nodes (0/34) and both ovary. Histologically, the para-aortic lymph nodal metastasis exhibited similar characteristics with the primary lesions described above, supporting the diagnosis of malignant uterine PEComa with para-aortic lymph nodal metastasis (Fig. 6 and 40×). The final stage was T3N1M0, where the Federation International of Gynecology and Obstetrics (FIGO) stage of uterine neoplasms was stage IIIC2.

The patient recovered smoothly and was discharged 18 days after the second surgery. Given the concerns of the large size of the tumor, the lymph nodal metastasis and other malignant features, the patient received 8 cycles of AI every three weeks starting from 1 week after the second surgery. The AI regiment prescribed was the intravenous administration of epirubicin (60 mg/m^2^,100–110 mg) on day 1 along with daily ifosfamide (4.5 g/m^2^in total, which was 1.5 g/m^2^/d) from day 1 to day 3(7–7.5 g in total, 2.5–3.0 g/d). Grade 1–2 drug-related toxic effects such as gastrointestinal reactions and myelosuppression were observed and closely monitored during the treatment. We also recommended everolimus, an mTOR inhibitor, to the patient for maintenance treatment after chemotherapy, but she failed to follow because of economic burden. Up to now, the patient has been free of recurrences or distant metastases for 6 years after surgery.

## Discussion and conclusions

Uterine PEComas were first documented in 2002 [6]. Increasing cases have been reported since then, likely linked to the improved cross-sectional imaging and histological/immunohistochemical tumor recognition in particular. With a combination of diagnostic methods, we herein described a case of uterine PEComa with typical pathological features such as epithelioid to spindle tumor cells exhibiting eosinophilic to clear cytoplasm, an intimate relationship with blood vessels (perivascular distribution), and immunoreactive for uterine PEComa markers including HMB-45, Melan-A, and Cathepsin K. It is noteworthy that most of the patients with uterine PEComas may experience abnormal uterine bleeding, lower abdominal pain with a palpable mass, and menstrual disorders, which are similar to the symptoms and signs of other uterine tumors and often lead to misdiagnosis before surgery. Not surprisingly, the case presented in this report was initially misdiagnosed as uterine leiomyoma before surgery and then as uterine leiomyosarcoma upon pathological examination following the first surgery at the local hospital. After careful review of the histologic slides and additional immunostaining of multiple markers, we confirmed the diagnosis of uterine PEComa.

The histologic and immunohistochemical features of PEComas suggest that these tumors might emerge from the vascular parietal structures even though neither a corresponding normal cellular counterpart nor a precursor lesion for PEComas has been identified [2, 7]. Given the rarity of PEComas, little is known with regards to their etiology, molecular profile, and risk factors of these tumors. Patients with PEComas have been frequently shown to bear mutations in the tuberous sclerosis complex (*TSC1*) and/or *TSC2* genes. The products of *TSC1* and *TSC2* gene form a heterodimer which negatively regulate mTOR pathway inhibiting cell proliferation and metabolism, and inactivation of these two genes results in increased cell growth and proliferation [8, 9]. In uterine PEComas, however, only ~ 10% of the cases are associated with Bourneville tuberous sclerosis [7, 10, 11].

Besides *TSC1* and/or *TSC2* mutations, a distinctive subset of PEComas which carry gene rearrangements involving transcription factor E3 (*TFE3*) translocation and fusion has also been identified [12]. Confirmed cases of *TFE3*-rearranged PEComas are only a few and the significance of *TFE3* rearrangements remains unclear. The clinicopathologic findings suggest that this subset of PEComas has a unique morphology and specific immunophenotype without *TSC1/2* mutations [13]. One documented case with cervical *TFE3*-rearranged PEComa has aggressive behavior and poor prognosis, further raising an concern regarding a potential association between *TFE3* rearrangements and PEComas malignancy [14].

Of interest, RAD51B-OPHN1 fusion, an even rarer gene rearrangement has been identified recently in 4 patients with uterine PEComas but not present at any other locations [15, 16]. Although the cohort of RAD51B-rearranged PEComas is small, preliminary data suggest this fusion is associated with aggressive behavior, but additional studies need to be performed to corroborate this observation.

Moreover, the vast majority of PEComas have been described in females, with a female-male ratio as high as 9:1 noted in some case studies, suggesting a possible role of hormones in the pathogenesis of the disease. Indeed, increased expression of ER and PR has been shown in the patients with uterine PEComas [17–19]. The case presented in this report also exhibited strong nuclear staining of ER and PR in both spindle and epithelioid tumor cells. Whether uterine PEComas are hormone-dependent disease worth further study, and the significance of ovary dissection in patient with increased expression of ER and PR is unclear. Bilateral oophorectomy was performed in the case presented in this report .

Although most PEComas follow a benign course, malignant cases are being increasingly reported in different organs including the uterus. The original classification established by Folpe et al. categorizes PEComas as either benign (no atypical features), uncertain malignant potential (nuclear atypia/multinuclear giant cells or size > 5 cm), or malignant (two or more of the following: size > 5 cm, infiltrative growth, high nuclear grade and cellularity, mitoses > 1/50 HPFs, necrosis, and lymphovascular invasion) [3]. Later on, a modified algorithm specific to gynecologic PEComas has been proposed to classify tumors as malignant (at least 4 worrisome features are present) and benign/uncertain malignant potential (fewer than 4 features) [20]. The aggressiveness of malignant PEComas also appears to be variable. For example, in a retrospective study with 36 malignant PEComa patients, 26 (72%) developed metastases, especially to the lungs (21.6%) and liver (17.6%), but also in the peritoneum (10.8%) or lymph nodes (9.5%) [11]. In our case, the size of the primary uterine tumor was about 7 to 8 cm in diameter, together with high mitotic figures, necrosis, local infiltration in the uterine wall, and lymph node metastasis, supported the diagnosis of malignant uterine PEComa according to the proposed criteria.

Due to the small number of reported cases and inconsistent results obtained from various therapeutic strategies, currently there is no consensus on the treatment of PEComas. Surgery is the main treatment of PEComas for primary lesions as well as for local recurrences and metastases. The extend of surgery should be individual regarding the health situation of the patients and the biological behavior of their tumors, with the aim to obtain tumor-free margins. Most PEComas appear to be curable with surgical resection alone, whereas the actual effect of adjuvant therapies including radio-, chemo-, and immunotherapy remains unclear. However, given the uncertain tumor biology of PEComas, postoperative radiotherapy and chemotherapy should be supplemented for patients with locally advanced or metastatic PEComas [5]. Moreover, as the pathogenesis of PEComas is usually associated with mutations in the *TSC1* and/or *TSC2* genes, targeted therapy using mTOR inhibitors seems to show encouraging results in a few studies [21–23]. Nevertheless, the therapeutic effects of mTOR inhibitors on PEComas warrant additional investigation in prospective studies as a case report has shown drug resistance in a patient receiving mTOR inhibitor treatment of metastatic PEComa [24]. In our case, after surgical resection of the primary tumor and metastatic lymph nodes, the patient received 8 cycles of chemotherapy with a combination of epirubicin and ifosfamide. Fortunately, even with multiple high risk features, the patient survived without recurrence and metastasis for a relatively long period (5 years up to now).

Notably in some cases, metastatic spread of PEComas may present many years after initial treatment [19, 25], which underscores the importance of long-term follow-up of patients with PEComas and also highlights the need for criteria to accurately predict the outcome of PEComas and to help guide management decisions.

## Data Availability

Data sharing is not applicable to this article as no datasets were generated or analysed during the current study.

## Abbreviations

S-100: Schwannoma marker
CK18: Epithelial marker cytokeratin 18
CT: Computed tomography
ER: Estrogen receptor
FIGO: Federation International of Gynecology and Obstetrics
MRI: Magnetic resonance imaging
PEComas: Perivascular epithelioid cell tumors
PR: Progesterone receptors
Syn: Endocrine marker synaptophysin
*TFE3*: Transcription factor E3
*TSC1*: Tuberous sclerosis complex
